# Analysis of hip joint loading during walking with different shoe types using instrumented total hip prostheses

**DOI:** 10.1038/s41598-021-89611-8

**Published:** 2021-05-12

**Authors:** Y. Palmowski, S. Popović, D. Kosack, P. Damm

**Affiliations:** 1 Center for Musculoskeletal Surgery, Charité - Universitätsmedizin Berlin, Corporate Member of Freie Universität Berlin, Humboldt-Universität Zu Berlin, and Berlin Institute of Health, Berlin, Germany; 2 Julius Wolff Institute, Berlin Institute of Health at Charité – Universitätsmedizin Berlin, Berlin, Germany

**Keywords:** Lifestyle modification, Rehabilitation

## Abstract

Hip joint loads need careful consideration during postoperative physiotherapy after joint replacement. One factor influencing joint loads is the choice of footwear, but it remains unclear which footwear is favorable. The objective of the present study was to investigate the influence of footwear on hip joint loads in vivo. Instrumented hip endoprostheses were used for in vivo load measurements. The parameters resultant contact force (F_res_), bending moment (M_bend_) and torsional moment (M_tors_) were evaluated during treadmill walking at 4 km/h with different shoe types. In general, footwear tended to increase hip joint loading, with the barefoot shoe having the least influence. F_res_ and M_bend_ were significantly increased during heel strike for all shoe types in comparison to barefoot walking, with everyday shoe (34.6%; p = 0.028 and 47%; p = 0.028, respectively) and men’s shoe (33.2%; p = 0.043 and 41.1%; p = 0.043, respectively) resulting in the highest changes. M_tors_ at AbsMax was increased by all shoes except for the barefoot shoe, with the highest changes for men’s shoe (+ 17.6%, p = 0.043) and the shoe with stiffened sole (+ 17.5%, p = 0.08). Shoes, especially those with stiff soles or elaborate cuishing and guiding elements, increase hip joint loads during walking. The influence on peak loads is higher for M_tors_ than for F_res_ and M_bend_. For patients in which a reduction of hip joints loads is desired, e.g. during physiotherapy after recent surgery or to alleviate symptoms of osteoarthritis, low profile shoes with a flexible sole may be preferred over shoes with a stiff sole or elaborate cushioning elements.

## Introduction

Biomechanical stress in the hip joint is widely recognized as a major factor contributing to the development of hip osteoarthritis^1^. Furthermore, hip joint loads may also influence the postoperative outcome of patients undergoing total hip arthroplasty as excessive joint loads may increase the risk of complications such as loosening or implant wear. Detailed knowledge about the underlying biomechanics, particularly regarding the forces acting on the hip joint in everyday life, is an essential requirement for the further improvement of these factors in order to achieve an optimal postoperative outcome. One potential factor influencing the hip joint loading during walking is the shoe type.

From an evolutionary point of view, the human musculoskeletal system is mainly adapted to barefoot walking on soft ground^2^. When shoes started to be commonly used, their role remained for a long time solely that of a protection against cold and injuries. Therefore, they were mainly made of fur or thin leather. Particularly during the past decades shoes have moved beyond their original purpose of mere protection, creating higher than ever demands regarding both their design and functionality. It has been shown that shoes affect joint loading in walking and running compared to barefoot conditions or compared to lighter and more flexible footwear^3–11^. As far as clinical applications are concerned, previous studies showed that walking 6 months in minimalist footwear relieved pain and decreased knee loading in subjects with knee osteoarthritis^9,10^. These findings have lead to an increasing trend “back to roots” during the past years, resulting in shoes with thin flexible soles devoid of any cushioning elements. On the other hand, in long-distance running shoes with curved carbon plates embedded between thick light-weight midsoles were shown to improve the energy cost of running (running economy) by 2.6–4% compared with track spikes and established marathon running shoes^3–6^. These can potentially lead to substantial improvements of running performances^7^. Athletes wearing versions of Nike’s Vaporfly dominate the long-distance running since the 2016 Rio Olympic Marathon and took 31 of the 36 podium places at the six marathon majors in 2019, thus leading to a clear shift regarding the way of current construction of long-distance performance running shoes^12^.

Despite the recent surge in general interest on this topic, the actual effect of footwear on forces and moments of the hip joint has not yet been adequately examined. It is still unkown if certain shoe types might help to reduce joint loads and thereby alleviate symptoms of osteoarthritis or if others might on the contrary aggravate symptoms and hinder the healing process after total joint arthroplasty by increasing joint loads. An increasing number of studies regarding joint loads during activities of everyday life and with different shoe types have been published in the last years, which however mainly rely on mathematical models^8,13–23^. These models use data from gait analyses to calculate internal joint loads. Due to the indirect nature of the measurements, the results remain controversial and do not allow definite statements. An alternative method that allows the direct determination of joint loads is the use of instrumented implants^24^. In this study, we aimed to analyse the influence of various common shoe types on the in vivo hip joint loading during walking in patients with instrumented total hip prostheses^25^. Thereby, we wish to derive new insights regarding which shoe types should be preferred for patients suffering from osteoarthritis of the hip as well as during the healing phase after total hip arthroplasty. Our hypothesis was that wearing footwear does not decrease joint loads of the hip as compared to barefoot walking.

## Materials and methods

### Ethics statement

The Ethics Committee of the Charité—Universitätsmedizin Berlin, Germany approved the implantation and the study protocols (EA2/057/09). All patients gave written informed consent prior to participation in these studies, in which they agreed to implantation of the instrumented implants, in vivo load measurements and the publication of their images. All methods were performed in accordance with the relevant guidelines and regulations.

### Instrumented implants

**Figure 1:**
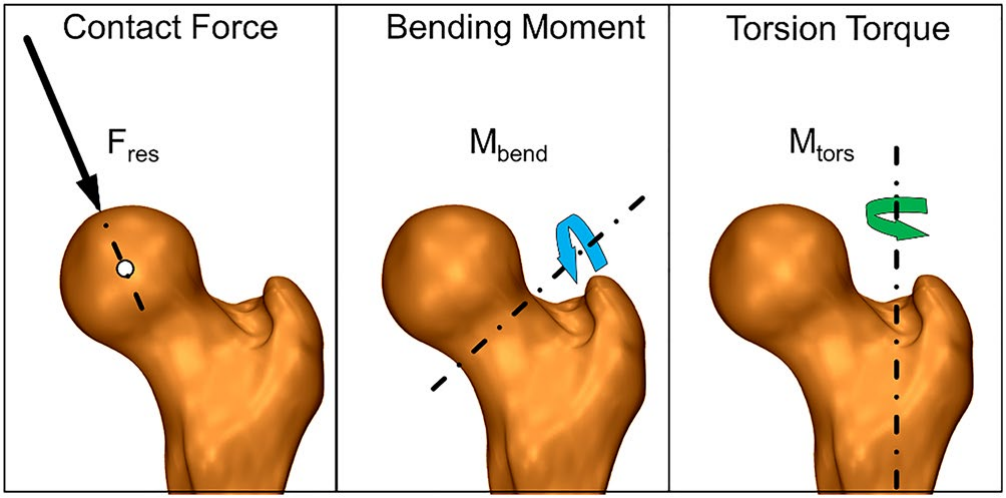
Localisation and direction of the parameters resultant contact force (F_res_), bending moment (M_bend_) and torsional moment (M_tors_).

In this study an instrumented hip endoprosthesis was used for in vivo load measurement^25^. The prosthesis is based on a clinically proven cementless implant (CTW, Merete Medical, Berlin, Germany) with a titanium stem and a 32 mm Al_2_O_3_ ceramic head. The implant was modified to house an inductive power supply, six strain gauges, signal amplifiers and telemetric data transmission in the hollow neck. The strain gauges were used to detect the load depending micro-deformations of the neck, which were transformed into three force and three moment components relative to the implant, by using an implant specific calibration respectively measurement matrix and subsequently transferred into an femur based coordinate system^26^. Details regarding the instrumented implants and the measurement accuracy have been published previously^25^. For the measurements of forces and moments, a femur-based coordinate system was centred at the head of a right-side implant. Data from the left side were mirrored. For the present study, the resultant contact force F_*res*_ acting at the femoral head, the bending moment M_*bend*_ acting in the middle of the femur neck as well as the torsion torque M_*tors*_ in the bone-stem-interface respectively in femur shaft axis (Fig. 1) were analyzed.

### Patients

Six patients with an instrumented hip implant participated in this study (Table 1). These patients are part of a collective of overall 10 patients with instrumented implants that has already been used for other studies, the results of which have been published previously^27–36^. To perform the reported sub-study here, all subjects of these worldwide unique patient cohort were asked again to attend and six of them agreed to participate on this in vivo load measurement (Table 1). Measurements were taken between 9 and 25 months postoperatively during treadmill walking with 4 km/h. This walking speed was chosen according to the average gait speed of the respective age group and taking into account the pre-existing medical condition of the hip joint in our collective^37^. All patients confirmed 4 km/h as a comfortable walking speed during tests before starting the measurements. All patients had received the total hip arthroplasty due to osteoarthritis.

**Table 1:** Patients participated; *M* mean, *SD* standard deviation, *BMI* body mass index.

Participants	Gender	Age [years]	Weight [kg]	Height [cm]	BMI [kg/m^2^]	Time since surgery [months]	Implant side
H2R	M	64	80	172	26.9	32	Right
H5L	W	64	87	168	30.9	25	Left
H6R	M	69	85	176	27.5	18	Right
H7R	M	54	92	179	28.7	17	Right
H8L	M	56	85	178	26.7	13	Left
H9L	m	55	119	181	36.3	9	Left
M ± SD		60 ± 6	91 ± 14	176 ± 5	30 ± 4	19 ± 8	

### Shoes

Six different shoe types were used within in this study.

*VIBRAM Five Fingers Bikila LS (barefoot shoe), Converse AS OX CAN (everyday shoe), ADIDAS Salvation 3 (sports shoe), RIEKER Antistress Luciano (men’s shoe), MBT-Masai-Barefoot-Technoloy, and a shoe with a stiffened sole.* The BIKILA LS barefoot shoe has a flat shape with a three millimeter thick polyurethane inner sole and a 4 mm thick outer sole. Distinctive feature of the shoe is its anatomical shape with separate toes. The Converse AS OX CAN everyday shoe has a textile mouth and a thin plastic sole, which offers little cushioning. The ADIDAS Salvation 3 is a flexible sports shoe which is supposed to adapt to the ground conditions at every step. The midfoot area contains a support system for a better roll-over and is stabilized against excessive pronation. The MBT has a special rounded sole construction and a flexible heel element in order to imitate walking on soft, natural ground and thereby create an instability. The Rieker Antistress Lucioano is a classical men’s shoe made of leather. For the shoe with a stiffened sole, the sole of a standard sneaker was stiffened to reduce flexibility. Additionaly information regarding the examined shoe types is available in Supplementary Table S1.

### Measurements

In order to collect the in vivo joint load data, the patients were asked to walk on a treadmill with 4 km/h. Before starting the measurements, the patients were given 5 min to get familiar with the respective shoes. Each measurement consisted of at least 30 ipsilateral step-repititions. Selected trials of each measurement are published and can be downloaded at the public data base www.OrthoLoad.com.

### Data collection and evaluation

During the measurements, the patients were videotaped and the image was recorded on video together with the telemetric in vivo load signals. Details on the external measurement system have been described previously^38^.

For better inter-individual comparability, in vivo measured forces and moments were given in percent of the bodyweight ([%BW] and [%BWm]). The time-load patterns of each patient were first averaged individually and for each shoe type separately using a time warping method^39^. Subsequently, the individual curves from the separate patients were averaged interindividually to calculate “shoe specific” time load patterns for each load component.

Finally, the curves were evaluated using defined points that are characteristic for the time load pattern during gait for the respective value. For F_res_ and M_bend_, these points were the ipsilateral heel-strike (HS) as well as the two local load maxima at the time of contralateral toe-off (CTO = 1. Max) and contralateral heel-strike (CHS = 2. Max). For M_tors_, the absolute maximum (AbsMax) at CTO was the characteristic point.

### Statistical analysis

Statistical analysis was performed using IBM SPSS Statistics 21.0 (New York, USA) and Microsoft Excel 2011 (Washington, USA). Mean values between all shoe types were compared regarding significant differences using Friedmann Test for non-parametric variables. In order to avoid multiple testing problems, direct comparisons between shoe types were only performed against barefoot walking. For this purpose, non-parametric Wilcoxon Test for paired samples was applied and differences were examined for significance using two-tailed tests with a significance level of p < 0.05. Relative differences between barefoot measurements and those with different shoe types were calculated on the basis of the mean values.

### Ethics approval

The Ethics Committee of the Charité—Universitätsmedizin Berlin, Germany approved this study (EA2/057/09). All participants gave written informed consent before data collection began.

## Results

### Resultant contact force F_res_

**Figure 2:**
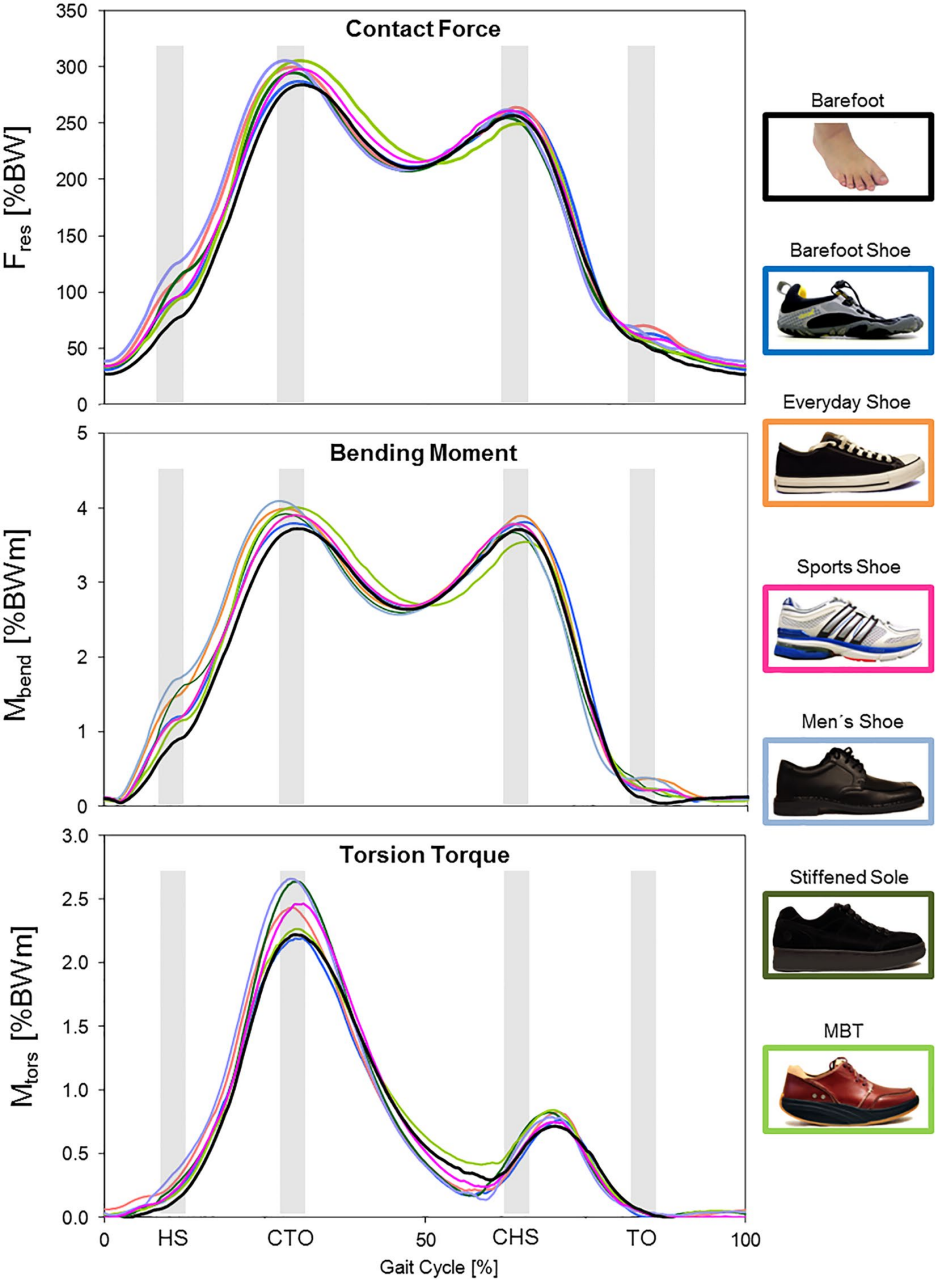
In vivo acting hip joint loads—resultant contact force (F_res_), bending moment (M_bend_) and torsional moment (M_tors_)—during walking with different shoes. *BW* body weight, *BWm* body weight meter.

F_res_ shows a pattern that is characteristic for walking (Fig. 2). The standing phase starts with the ipsilateral heel strike (HS), which is followed by two maxima at the time contralateral toe-off (CTO) and contralateral heel strike (CHS). The end of the standing phase is marked by the ipsilateral toe off (ITO). The Friedmann Test confirmed significant differences in joint loads between the various shoe types (Supplementary Table S2). HS shows an increased variability with barefoot walking resulting in the lowest contact force in the hip joint (102.6%BW) while all shoes lead to an increase in F_res_ (Table 2). The first maximum CTO as well shows an increased variability with barefoot walking resulting in the lowest contact force, but is only significantly increased by specific shoe types (men’s shoe, MBT and shoe with a stiffened sole). The second maximum CHS shows a smaller variability without a relevant influence of the shoe type (between 250.3%BW and 264.5%BW) and is only significantly increased by the men’s shoe. The percental difference in F_res_ between barefoot walking and the respective shoe types is presented in Table 2 and ranges from + 20% to + 34.6% at HS and from + 1.1 to + 8.0% at CTO.

**Table 2:** Deltas [%] of the resultant contact force F_res_ in relation to barefoot walking at 4 km/h; *HS* heel strike, *SD* standard deviation, *MBT* Masai-Barefoot-Technology.

	HS (± SD)	Δ [%]	p	1. Max (± SD)	Δ[%]	p	2. Max (± SD)	Δ [%]	p
Barefoot	102.6 (± 35.4)	n.a	n.a	284.3 (± 22)	n.a	n.a	257 (± 37.6)	n.a	n.a
Barefoot-shoe	123.1 (± 39.7)	+ 20.0	0.046	287.3 (± 25.8)	+ 1.1	0.463	260.1 (± 37.5)	+ 1.2	0.917
Everyday shoe	138.1 (± 36.7)	+ 34.6	0.028	300.1 (± 38.4)	+ 5.6	0.116	264.5 (± 35.6)	+ 2.9	0.463
Men’s shoe	136.7 (± 40.0)	+ 33.2	0.043	305.1 (± 25.5)	+ 7.3	0.043	261.7 (± 40.8)	+ 1.8	0.043
MBT	123.2 (± 37.8)	+ 20.1	0.028	307 (± 32.6)	+ 8.0	0.028	250.3 (± 43.6)	− 2.6	0.463
Sports shoe	124.7 (± 35.8)	+ 21.5	0.028	298.3 (± 30.6)	+ 4.9	0.075	261.3 (± 38.4)	+ 1.7	0.753
Stiffened sole	128.4 (± 28.6)	+ 25.1	0.043	294.9 (± 21.1)	+ 3.9	0.043	254.9 (± 40.8)	− 0.8	0.138

### Bending moment M_bend_

The bending moment (M_bend_) acting in vivo in the femur neck generally shows a pattern similar to that of F_res_ (Fig. 2). Same as for F_res_, M_bend_ appears to be overall lowest when walking barefoot and shows the greatest variability at HS and CTO. Again, all examined shoes lead to a significantly increased load at the time of HS (Table 3) between 1.7%BWm and 2.0%BWm as compared to 1.3%BWm for barefoot walking. The highest increase was observed for the everyday shoe with a delta of + 47% (Table 3). At CTO there were no significant differences between shoe types. Only everyday shoe and men’s shoe lead to a significant increase at CHS of 5.2% and 2.1%, respectively.

**Table 3:** Deltas [%] of the bending moment M_bend_ in relation to barefoot walking at 4 km/h; *HS* heel strike, *SD* standard deviation, *MBT* Masai-Barefoot-Technology.

	HS (± SD)	Δ [%]	p	1. Max (± SD)	Δ [%]	p	2. Max (± SD)	Δ [%]	p
Barefoot	1.3 (± 0.6)	n.a	n.a	3.9 (± 0.6)	n.a	n.a	3.7 (± 0.6)	n.a	n.a
Barefoot-shoe	1.7 (± 0.8)	+ 26.0	0.028	3.8 (± 0.8)	− 1.6	0.917	3.8 (± 0.6)	+ 2.5	0.463
Everyday shoe	2.0 (± 0.8)	+ 47.0	0.028	4.0 (± 0.9)	+ 3.7	0.463	3.9 (± 0.7)	+ 5.2	0.028
Men’s shoe	1.9 (± 0.8)	+ 41.1	0.043	4.1 (± 0.9)	+ 6.0	0.345	3.8 (± 0.8)	+ 2.1	0.043
MBT	1.7 (± 0.8)	+ 23.8	0.046	4 (± 0.8)	+ 3.4	0.345	3.5 (± 0.7)	− 4.8	0.075
Sports shoe	1.7 (± 0.7)	+ 26.3	0.028	3.9 (± 0.8)	+ 1.3	0.463	3.8 (± 0.7)	+ 2.1	0.173
Stiffened sole	1.8 (± 0.7)	+ 31.2	0.043	3.9 (± 0.8)	+ 1.5	0.5	3.7 (± 0.7)	− 1.2	0.345

### Torsional moment M_tors_

The torsional moment M_tors_ acting in the femur shaft respectively in the stem-bone-interface shows a clear maximum at CTO, corresponding to the first maxima of M_bend_ and F_res_ (Fig. 2). A further smaller maximum occurs shortly after CHS. Unlike M_bend_ and F_res_, there is no initial maximum at HS. The influence of the individual shoe type on the joint load is highest at the absolut maximum, where we see the highest variability. All shoes except the barefoot shoe tend to increase M_tors_ compared to barefoot walking (Table 4), but only sports shoe and men’s shoe result in significant differences at M_tors_ at HS with a delta of + 10.2% and + 17.6%, respectively.

**Table 4:** Torsion moment M_tors_ and the deltas [%] in relation to barefoot walking at 4 km/h; *AbsMax* absolute maximum, *SD* standard deviation, *MBT* Masai-Barefoot-Technology.

	AbsMax. (± SD)	Δ [%]	p
Barefoot	2.3 (± 0.2)	n.a	n.a
Barefoot-shoe	2.3 (± 0.3)	− 0.5	0.917
Everyday shoe	2.5 (± 0.5)	+ 7.5	0.345
Men’s shoe	2.7 (± 0.2)	+ 17.6	0.043
MBT	2.4 (± 0.4)	+ 4.5	0.6
Sports shoe	2.5 (± 0.2)	+ 10.2	0.046
Stiffened sole	2.7 (± 0.4)	+ 17.5	0.08

## Discussion

In this study the individual in vivo hip joint loads during walking at different speeds and with different shoe types were determined in six patients. For this purpose, we used instrumented implants, which allowed us to conduct direct measurements of the respective joint loads in vivo. When comparing our data to existing studies it needs to be kept in mind that most studies calculate the internal forces from external measurement using the mathematical multibody models. Even though methods such as rigid body musculoskeletal dynamic simulations are able to predict intrisicly generated forces like muscle forces, they do not directly measure them and may thereby come to different results.

Regarding the influence of different shoe types during level walking, we observed a significant increase in F_res_ and M_bend_ at heel contact (HS) for all shoe types. The resultant contact force (F_res_) measured in vivo increased between 20 and 35% with the highest values wearing the everyday shoe. This shoe does not have any special cushioning or guiding features The lowest increase was measured for the ‘barefoot shoe’, which is as well devoid of any cushioning elements, but in addition has a very flexible sole. A majority of studies has shown that barefoot walking leads to an increased plantarflexion, which enhances the physiological cushioning features of the foot arch and the ankle^11,40–42^. Previous studies have shown that an increased plantarflexion reduces the pressure at the hindfoot while increasing it in the forefoot area, which probably reduces the impact load at HS^43–45^. For the MBT, the increase in F_res_ at HS was almost as low as that of the barefoot shoe, supposedly due to its rounded sole shape that also causes an increased plantarflexion of the foot at the moment of ground contact and a faster motion transfer from dorsiflexion to plantarflexion. In comparison to the changes at HS, the relative increases in F_res_ for the 1 and 2 maximum were rather low, ranging between − 2.6 and 8%. Hence, the influence of the footwear on the maximum contact force appears to be quite small. Still, shoes generally had a tendency to increase joint load.

Similar to F_res_, the everyday shoe also showed the highest increase for M_bend_ and the MBT the smallest. Again, the first and second load maxima, which occur at CTO and CHS, only showed subtle changes of − 4.8% to 6%.

M_tors_ showed the highest sensitivity to the influence of shoe types during walking. This is of particular relevance as torsion moments are often considered to be more critical for implant loosening than contact forces. Except for the ‘barefoot shoe’, all examined shoe types lead to an increase of the absolute maximum of 4.5% to 17.6%. The men’s shoe and the shoe with stiffened sole resulted in the highest increase, possibly due to the the limited flexibility of the sole. This is in keeping with the results of a previous study using instrumented implants in a single patient, which came to the conclusion that very hard soles are not advisable^16^. Interestingly, sports shoes with special guiding and cushioning elements had a tendency for higher load increases (+ 10.2%) than a simple everyday shoe with a flat rubber sole (+ 7.5%). Several studies have already shown that guiding elements can force the foot in particular movement patterns, to which the locomotor system reacts with higher muscular activity and thereby increased the external joint moments^8,20,40,46^. Additionally, cushioning elements can hinder the proprioception of the foot sole and cause a certain instability at the time of ground contact that needs to be compensated by increased muscular acitivity^47,48^. The barefoot shoe on the other hand has shown the smallest influence, likely because it hardly reduces range of motion and proprioception. Additionally, it has a very thin sole, resulting in the smallest moment arms of vertical and mediolateral ground reaction force^8,49^. It is known that in shod conditions the eversion moment is higher due to a larger moment arm resulting from the increased width of the shoe and the heel flare^50^.

In general our findings confirm the results of previous studies that also showed decreased loadings on lower extremity joints for barefoot walking as compared to walking with footwork based on indirect mathematical models^22,51^. Therefore, our results suggest that low profile shoes with flexible soles might be preferable over those with elaborate cushioning elements for patients suffering from osteoarthritis or in order to minimize the stress on orthopaedic implants and their bone interfaces, which might help to reduce the risk of postoperative complications such as aseptic loosening.

Little is known so far as to which mechanical parameters play the most important role in aseptic implant loosening. A cadaveric study has shown loosening for non-cemented implant stems at a mean torsional movement of 33 Nm^52^. On the basis of the average patient weight of 91.3 kg in our study, this would correspond to 3.7%BWm for our collective. Although these in vitro data cannot be directly transferred to real life, it can be noted that such values were not seen for any of the examined shoe types in our study. Still, the increased stress caused by certain shoe types might be considered when deciding upon recommendations for operated patients, particularly in direct postoperative phase before complete osseous implant integration.

Another possible consequence of increased joint loads is the acceleration of implant wear. Especially the contact force seems to play in important role for the wear rate as it has an important influence on the tribological behaviors of hip prosthesis^53^. An increase in contact force directly results in a rise of the friction force^29,36^. Additionally, the contact force also has indirect impact on friction in the hip joint by influencing the thickness of the lubrication film, which is of high importance for friction reduction^54,55^. Several studies suggest that a higher contact force in the hip joint may increase implant wear rates^55–59^. However, in vivo data are still scarce and the effect also strongly depends on the materials of the respective surfaces, so that no specific statements are possible. As the influence of the shoe type on the maximum contact force was generally low, it seems unlikely that any of the examined shoe types has a relevant impact on implant wear.

The participants of our study were asked to perform the gait with unfamiliar footwear and only little time to get accustomed, so that we could only evaluate the immediate effect. It might be conceivable that joint loads change over time as the patients adapt their gait pattern to the shoes. A previous study examined the effect of laterally wedged shoes on knee joint loads and found similar results at baseline and after 4 weeks^60^. This suggests that the influence of gait adaptations in response to new footwear is overall low and would not have relevant impact on our results either.

Although our study showed a clear trend of increased joint loads for footwear, it is still limited by the small number of participants. A validation of our results in larger populations would be desirable, but seems difficult to realize due to the complex methodology. As a consequence of the rather small number of participants, it is possible that we were not able to detect all actual differences between the shoe types due to a lack of statistical power. While a larger cohort might help to identify additional differences between the various shoe types, the rather small sample size of the present study does not reduce the validity of the significant differences we were able to show. Also, all measurements rely on the same type of implant. Again, this is due to the methodology, but it should be noted that the geometry of the THA itself (e.g. offset) and surgical aspects such as the orientation of the implant (e.g. anteversion) may influence hip joint loads as well^61–63^. In this study, we focused on the influence of footwear on hip joint loads during walking, where forces are generally rather low in comparison. In clinical practice, changes during more demanding exercises like jogging might sometimes be more relevant, but have not been examined in this study. Moreover, our study only investigated the influence of footwear on the hip joint, whereas the effects on other joints of the lower extremity might be different.

As a conclusion, we could confirm the results from previous indirect studies and demonstrate that the in vivo hip joint load is smallest without shoes or with barefoot shoes. For patients in which a reduction of hip joints loads is desired, e.g. during physiotherapy after recent surgery or to alleviate symptoms of osteoarthritis, shoes with “well-intentioned” elaborate cushioning elements might actually be counterproductive. Quite the contrary, less sems to be more when it comes to joint-friendly footwear and low profile shoes with a flexible sole should be preferred.

## Supplementary Information

Supplementary Information.
