# Supplementary material for: Analysis of hip joint loading during walking with different shoe types using instrumented total hip prostheses
Source: Sci Rep. 2021 May 12;11:10073. doi: 10.1038/s41598-021-89611-8 (PMC8115277; doi:10.1038/s41598-021-89611-8)
Supplement: Supplementary file 1 — Supplementary Information. [file 41598_2021_89611_MOESM1_ESM.docx]

**Analysis of hip joint loading during walking with different shoe types using instrumented total hip prostheses**

Y. Palmowski^a^, S. Popović^b^, D. Kosack^b^, P. Damm^b^*

1. Center for Musculoskeletal Surgery, Charité - Universitätsmedizin Berlin, corporate member of Freie Universität Berlin, Humboldt-Universität zu Berlin, and Berlin Institute of Health, Berlin Germany
2. Julius Wolff Institute, Charité - Universitätsmedizin Berlin, corporate member of Freie Universität Berlin, Humboldt-Universität zu Berlin, and Berlin Institute of Health, Berlin Germany

**Corresponding author:*

Dr.-Ing. Philipp Damm

Julius Wolff Institut

Charite – Universitätsmedizin Berlin

Yannick Palmowski, Dr. med. yannick.palmowski@charite.de

Srđan Popović, Dr. phil. srdan.popovic@charite.de

Denise Kosack, Ing. kosack.denise@gmail.com

*Philipp Damm, Dr.-Ing. philipp.damm@charite.de

**Supplementary Table 1:** Properties of the examined shoe types

| **Shoetype / Qualities** | **Vibram Five Fingers Bikila LS** | **Converse As OX Can** | **Adidas Salvation 3** | **Rieker Antistress Luciano** | **Masai Barefoot Technology** | **Sneaker with a stiffed sole** |
| --- | --- | --- | --- | --- | --- | --- |
| sole material and special properties | rubber (Vibram Megagrip) | Vulcanized rubber | three-dimensional support of the foot against over-pronation | sole with shock absorbing material, anatomical foot bed | curved sole for defined rolling with shock absorbing effect and a provoked instability during gait, anatomical PU-insole | rubber, anatomical foot bed |
| upper material | synthetic/mesh | firm sail cloth,  toe cap: rubber | air Mesh-Nylon,  synthetic leather-overlay | leather | leather | leather |
| inner material | textil | textil, upholstered foot bed | removable insoles, textile/foam,  GeoFit ® technology lining | leather and textil | textil | textil |
| weight [g] | 130 | 150 | 364 | 340 | 650 | 520 |

|  | **F_res_** | | | | | | **M_bend_** | | | | | | **M_tors_** | |
| --- | --- | --- | --- | --- | --- | --- | --- | --- | --- | --- | --- | --- | --- | --- |
|  | HS | | 1. Max | | 1. Max | | HS | | 1. Max | | 1. Max | | AbsMax | |
|  | Mean | SD | Mean | SD | Mean | SD | Mean | SD | Mean | SD | Mean | SD | Mean | SD |
| Barefoot | 106.5 | 38.1 | 276.9 | 13.9 | 249.4 | 36.5 | 1.4 | 0.7 | 3.8 | 0.6 | 3.6 | 0.6 | 2.3 | 0.2 |
| Barefoot-shoe | 128.3 | 42.1 | 281.3 | 23.8 | 256.5 | 40.5 | 1.7 | 0.9 | 3.8 | 0.9 | 3.8 | 0.7 | 2.3 | 0.4 |
| Everyday shoe | 140.2 | 40.6 | 288.6 | 29 | 259.2 | 37.1 | 2 | 0.8 | 3.9 | 0.9 | 3.8 | 0.7 | 2.4 | 0.5 |
| Men’s shoe | 136.7 | 40 | 305.1 | 25.5 | 261.7 | 40.8 | 1.9 | 0.8 | 4.1 | 0.9 | 3.8 | 0.7 | 2.7 | 0.2 |
| MBT | 127.8 | 40.3 | 295 | 16.1 | 243.5 | 45.1 | 1.7 | 0.9 | 3.9 | 0.8 | 3.4 | 0.7 | 2.4 | 0.4 |
| Sports shoe | 125.6 | 39.9 | 288.1 | 19.7 | 253.7 | 37.5 | 1.7 | 0.8 | 3.8 | 0.8 | 3.7 | 0.7 | 2.5 | 0.3 |
| Stiffened sole | 128.4 | 28.6 | 294.9 | 21.1 | 254.9 | 40.8 | 1.7 | 0.6 | 3.9 | 0.8 | 3.7 | 0.7 | 2.7 | 0.4 |
| **p-value** | **0.013** | | **0.011** | | **0.160** | | **0.013** | | **0.044** | | **0.014** | | **0.021** | |

**Supplementary Table 2:** Friedmann Test for differences in joint loads between the different shoe types; HS: heel strike, SD: standard deviation, MBT: Masai-Barefoot-Technology
